# Degeneration of saccular hair cells caused by MITF gene mutation

**DOI:** 10.1186/s13064-019-0126-0

**Published:** 2019-01-11

**Authors:** Yi Du, Li-li Ren, Qing-qing Jiang, Xing-jian Liu, Fei Ji, Yue Zhang, Shuo-long Yuan, Zi-Ming Wu, Wei-Wei Guo, Shi-Ming Yang

**Affiliations:** Beijing Key Laboratory of Hearing Impairment Prevention and Treatment, Key Laboratory of Hearing Impairment Science, Chinese PLA Medical School, Beijng, China

**Keywords:** *MITF-M*, Cochleosaccular degeneration, Hearing loss, Waardenburg syndrome, Intermediate cell, Pig

## Abstract

**Background:**

Waardenburg syndrome (WS) is the consequence of an inherited autosomal dominant mutation which causes the early degeneration of intermediate cells of cochlear stria vascularis (SV) and profound hearing loss. Patients with WS may also experience primary vestibular symptoms. Most of the current WS studies did not discuss the relationship between WS and abnormal vestibular function. Our study found that a spontaneous mutant pig showed profound hearing loss and depigmentation. MITF-M, a common gene mutation causes type WS which affect the development of the intermediate cell of SV, was then identified for animal modeling.

**Results:**

In this study, the degeneration of vestibular hair cells was found in pigs with MITF-M. The morphology of hair cells in vestibular organs of pigs was examined using electron microscopy from embryonic day E70 to postnatal two weeks. Significant hair cell loss in the mutant saccule was found in this study through E95 to P14. Conversely, there was no hair cell loss in either utricle or semi-circular canals.

**Conclusions:**

Our study suggested that MITF-M gene mutation only affects hair cells of the saccule, but has no effect on other vestibular organs. The study also indicated that the survival of cochlear and saccular hair cells was dependent on the potassium release from the cochlear SV, but hair cells of the utricle and semi-circular canals were independent on SV.

## Background

The balance system mainly relies on vestibular organs which provides an internal reference of our positions [1]. Hair cells are the only sensory cells in vestibular organs which can detect linear or rotational head movement. It is crucial to notice that in mammals vestibular organs are formed during embryonic development and the damage of hair cell in those organs is irreversible [2, 3]. Vestibular hair cells loss caused by aging, genetic defects, or ototoxic drugs, can lead to significant balance disorders [4]. Impaired vestibular function is embodied in anomalous oculomotor, gazing, and postural responses during movements [1].

**Fig. 1:**
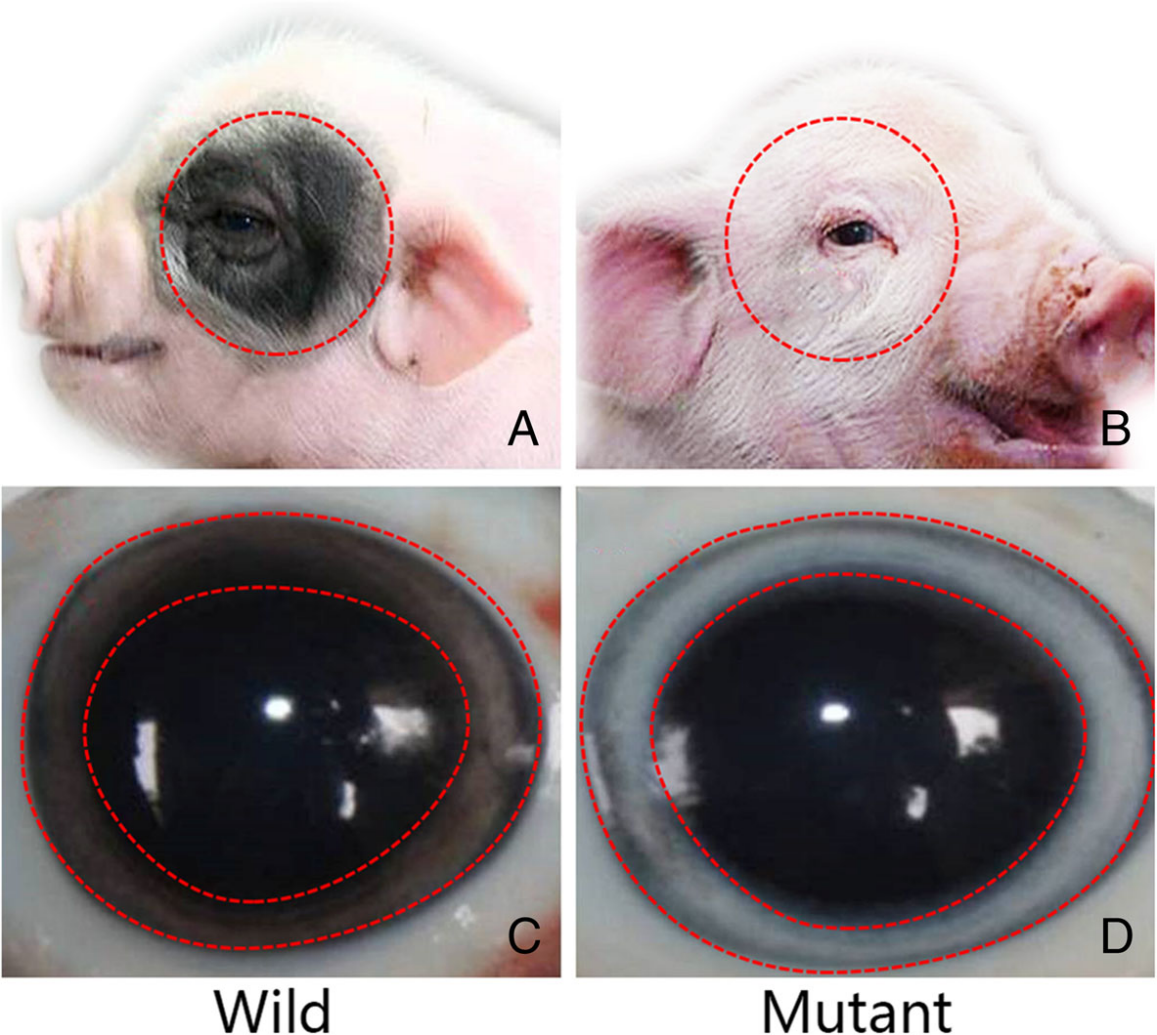
Hypopigmentation of fur, skin, and iris in MITF gene mutant pigs. (**a**) and (**c**) Wild-type miniature pig, (**b**) and (**d**) MITF-mutant type miniature pig

Animal models are important tools to study the mechanisms of vestibular hair cell degeneration and to evaluate their influence on vestibular function. Rongchang pig, known as “panda-pig,” is characterized by their black ring around their eyes. Our previous work reported a phenotype of miniature pigs with deafness and depigmentation which was caused by a mutation in the region of the melanocyte-specific promoter of microphthalmia-associated transcription factor (MITF) gene [5]. This expression of MITF-M isoform resembled the typical phenotype of Waardenburg syndrome type II (WS2) in humans, which was featured by severe hearing loss, heterochromia iridis and white forelocks [6, 7]. In this study, we depicted the same porcine model which exhibits WS2-like phenotypes, including depigmentation in the skin, fur, and iridis, spontaneous deafness as well as saccular hypofunction (Fig. 1). Studies related to cochleosaccular degeneration discussed its influence in a variety of animal models (mouse [8],cats [9], dogs [10, 11], mink [12]) and human [13].

Previous studies reported balance problems in patients diagnosed with WS. Marcus et al. studied vestibular testing results of twenty-two WS patients and summarized a common disturbed vestibular function [14]. Other studies by Black [15], Zelig [16], Stoller [17], Hagemen and Delleman [7], and Hildesheimer [18], also discovered abnormalities of vestibular function in WS patients. However, Hageman studied 25 WS patients noticing no apparent difference with other congenital hearing loss subjects. Besides, Hageman suggested that vestibular dysfunction should not be considered as an essential symptom in WS [7]. Thorkilgaard et al. also reported normal vestibular functions in WS patients [19]. Therefore it is still unclear whether the abnormal vestibular function should be considered as one of the characteristic features in WS patients.

Few studies have tested the effect of MITF-M gene mutation, a known gene mutation which causes WS, on vestibular function. Hence, in this study, we evaluated the morphological features of vestibular organs in MITF-M mutation animals.

## Results

### Morphological performance in MITF-mutation pig and wild type pig

#### Micro-CT scanning and 3D reconstruction

**Fig. 2:**
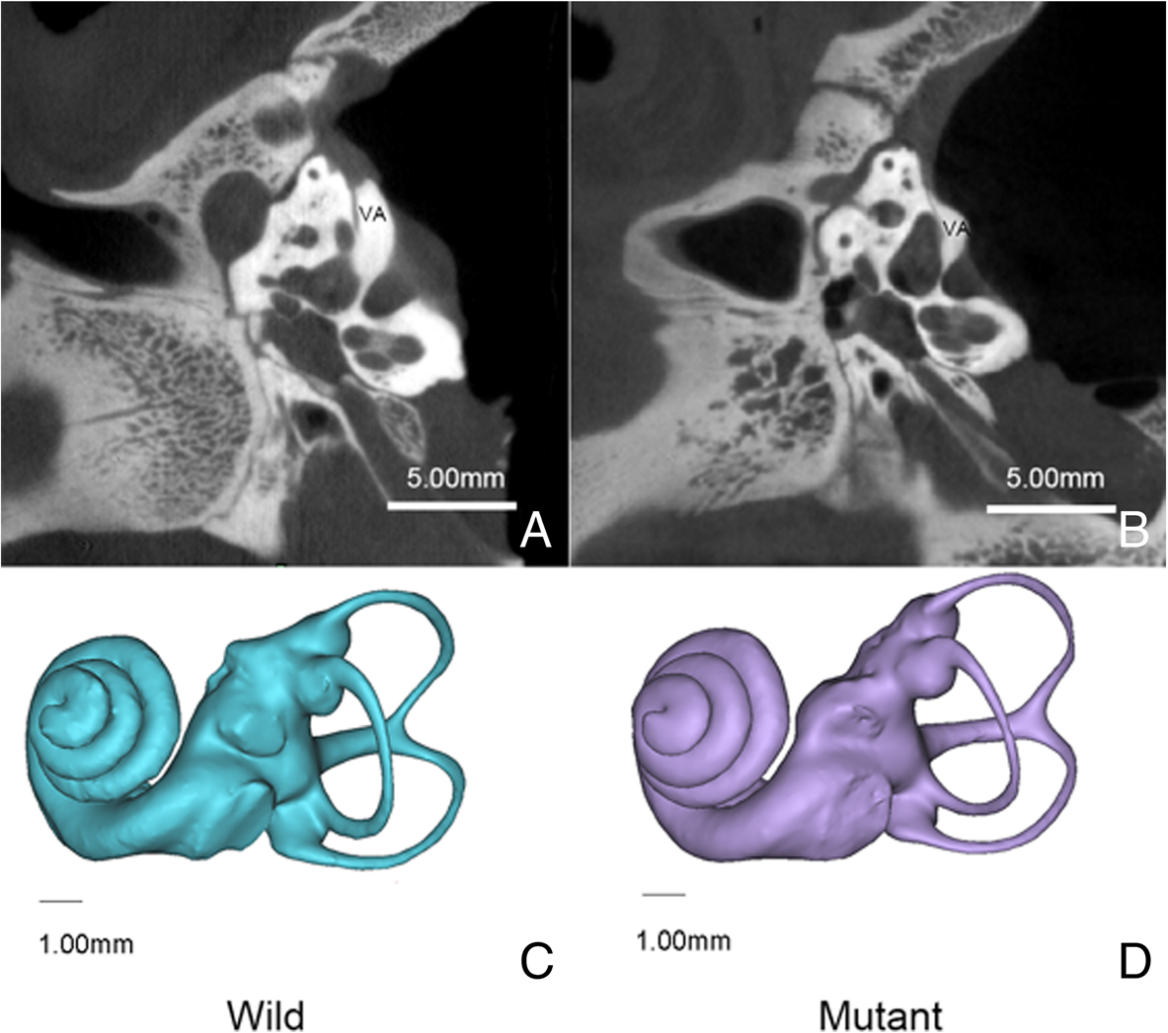
**a** and **b**: CT scans showed that the vestibular bony labyrinth. No difference was found between the WT (**a**) and albino pigs (**b**) (VA: vestibular aqueduct). **c** and **d**: Reconstructed left bony labyrinths of miniature pigs (**a**: wild miniature pig; **b**: MITF mutation miniature pig). Scale bars in **a** & **b** = 5 mm, in **c** & **d** = 1 mm

Micro-CT scanning of both the WT and albino pigs were performed. After comparing the CT images, no significant difference was observed between the WT and albino pigs. Labyrinth in all five vestibular organs showed no dysplasia, neither did the vestibular aqueduct, especially no dilation (Fig. 2a and b).

Based on the reconstructed 3D models of inner ears, whole spatial structures of the cochlea, vestibule, and semicircular canals were found in the WT and albino pigs (Fig. 2c and d). The morphological comparison between the WT and albino pigs showed no prominent dissimilarity. The three semicircular canals were placed in orthogonal positions and formed approximately 90 degrees between each other.

#### SEM results of vestibular systems

**Fig. 3:**
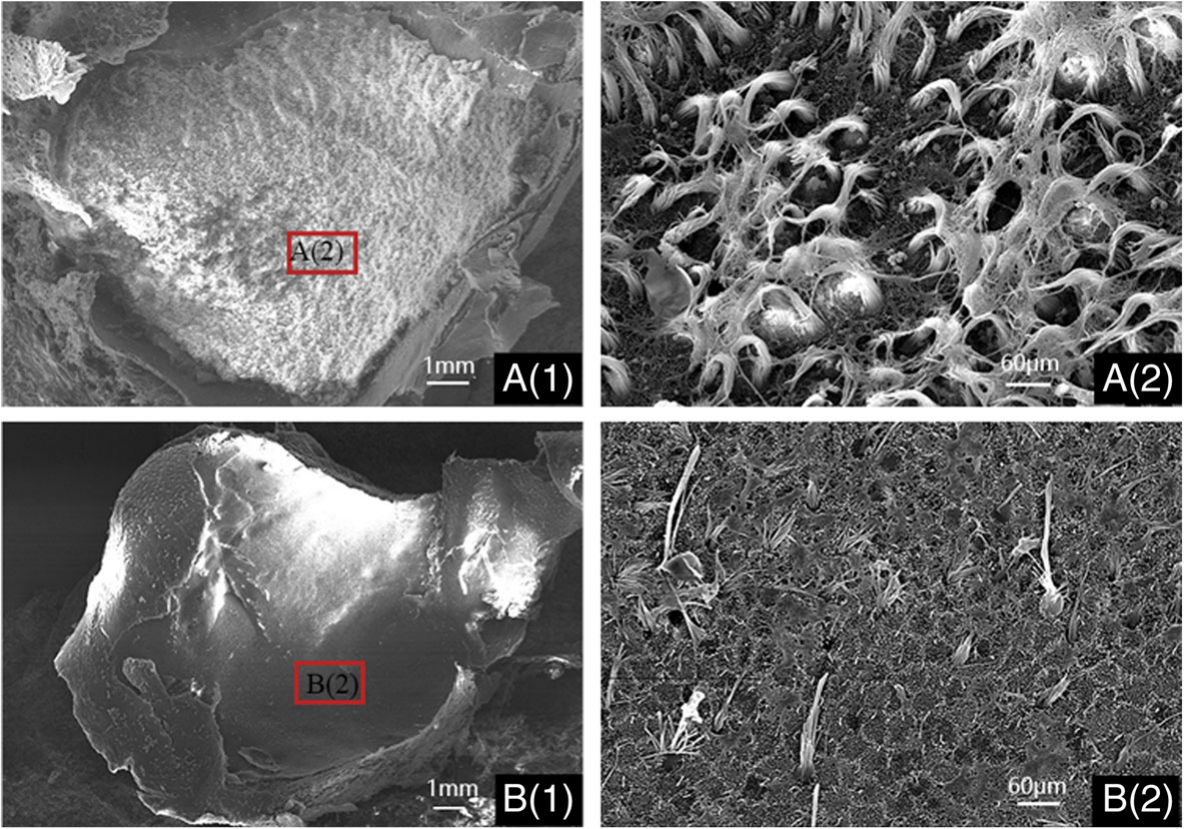
MITF-M mutation leads to the loss of saccular hair cells in miniature pigs (postnatal 14 days). **a** (1) Low magnification image of the saccule from wild miniature pig with numerous hair cells throughout the sensory epithelium; **a** (2) high magnification image of the saccular region from wild miniature pig (otoconia was removed for a better view); **b** (1): low magnification image of the saccule from MITF-mutation porcine model with extensive hair cell loss throughout the sensory epithelium; **b** (2): high magnification image of the saccular region from MITF-mutation porcine model. Scale bars in **a** (1) & **b** (1) = 1 mm, in **a** (2) & **b** (2) = 60 μm

The comparison of saccular hair cells in the WT and albino pigs was well described in Fig. 3. Saccular hair cells in the WT miniature pigs were well developed and all countable (Fig. 3a (1–2), otoconia was removed for a better view). By contrast, marked hair cell loss was found in albino pig (Fig. 3b (1–2)). The otoconia were absent, and the stereocilia appeared sparse and atypical, even barely existed in the mutant saccule. These results of SEM suggested extensive hair cell loss in saccule was associated with MITF-M gene mutation.

**Fig. 4:**
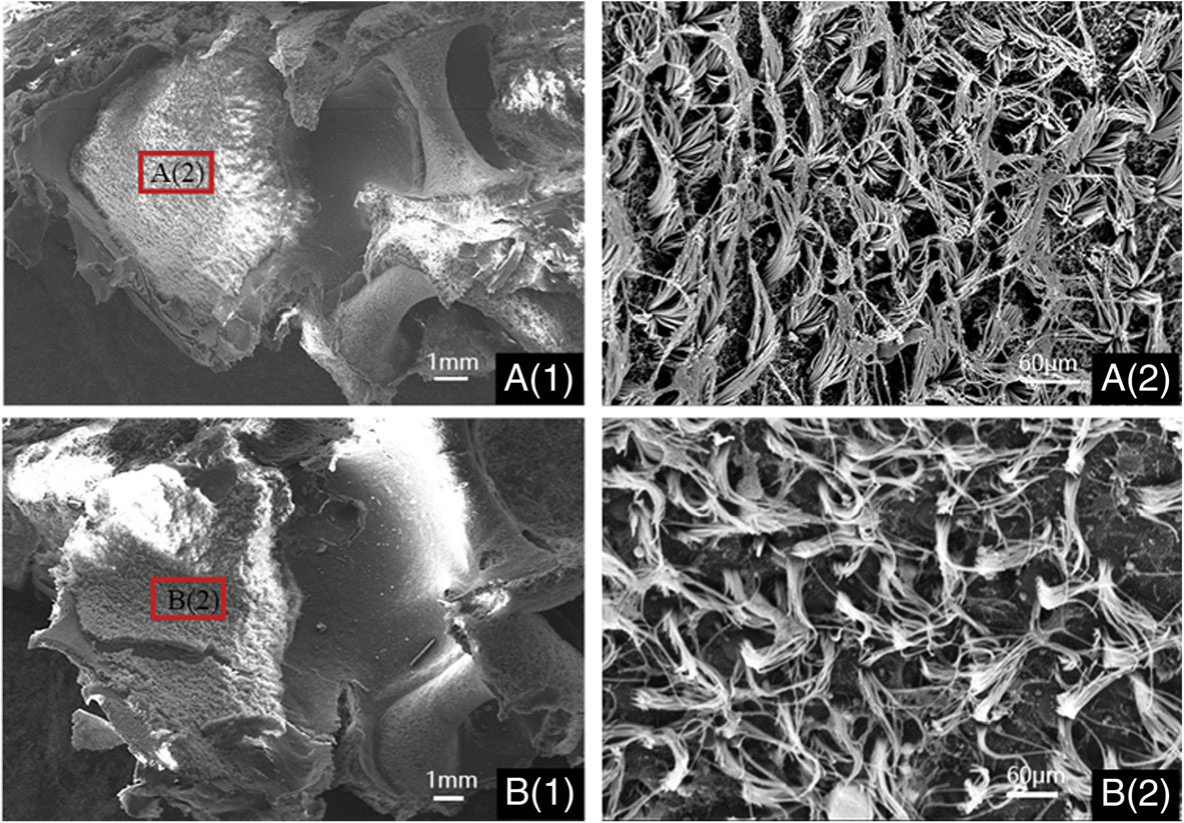
Scanning electron micrographs of utricles of postnatal 14 days old miniature pigs (otoconia was removed for a better view). **a** (1): low magnification image of the utricle from wild miniature pig with numerous hair cells throughout the sensory epithelium; **a** (2): high magnification image of the utricle region from wild miniature pig; **b** (1): low magnification image of the utricle from MITF-mutation porcine model with numerous hair cells throughout the sensory epithelium; **b** (2): high magnification image of the utricle region from MITF-mutation porcine model. Scale bars = 1 mm in **a** (1) & **b** (1). Scale bars = 60 μm in **a** (2) & **b** (2)

A thick layer of otoconia covered the utricle epithelial surface of both WT and albino pigs. (Fig. 4, otoconia was removed for a better view) No significant decrease of otoconia in utricle of the albino pig was observed compared with the WT pig.

**Fig. 5:**
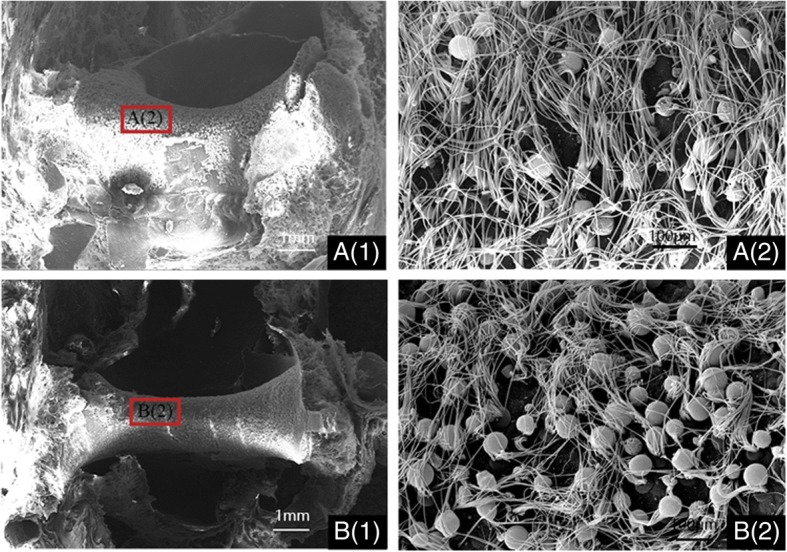
Scanning electron micrographs of the horizontal crista ampullas in postnatal 14 days miniature pigs. **a** (1): low magnification image of the crista from wild miniature pig with numerous hair cells throughout the sensory epithelium; **a** (2): high magnification image of the central apical region from wild miniature pig; **b** (1): low magnification image of the crista from MITF-mutation porcine model with numerous hair cells throughout the sensory epithelium; **b** (2): high magnification image of the central apical region from MITF-mutation porcine model. Scale bars = 1 mm in **a** (1) & **b** (1) and, 100 μm in **a** (2) & **b** (2)

Hair cells in the crista ampulla of horizontal canals in both the WT and albino pigs were shown in Fig. 5. No obvious pathology in hair cells was observed in the crista ampulla between the WT and mutant pigs. There was no difference in hair cell density of crista ampullas, which suggested MIFT-M mutation did not affect hair cells in ampulla.

**Fig. 6:**
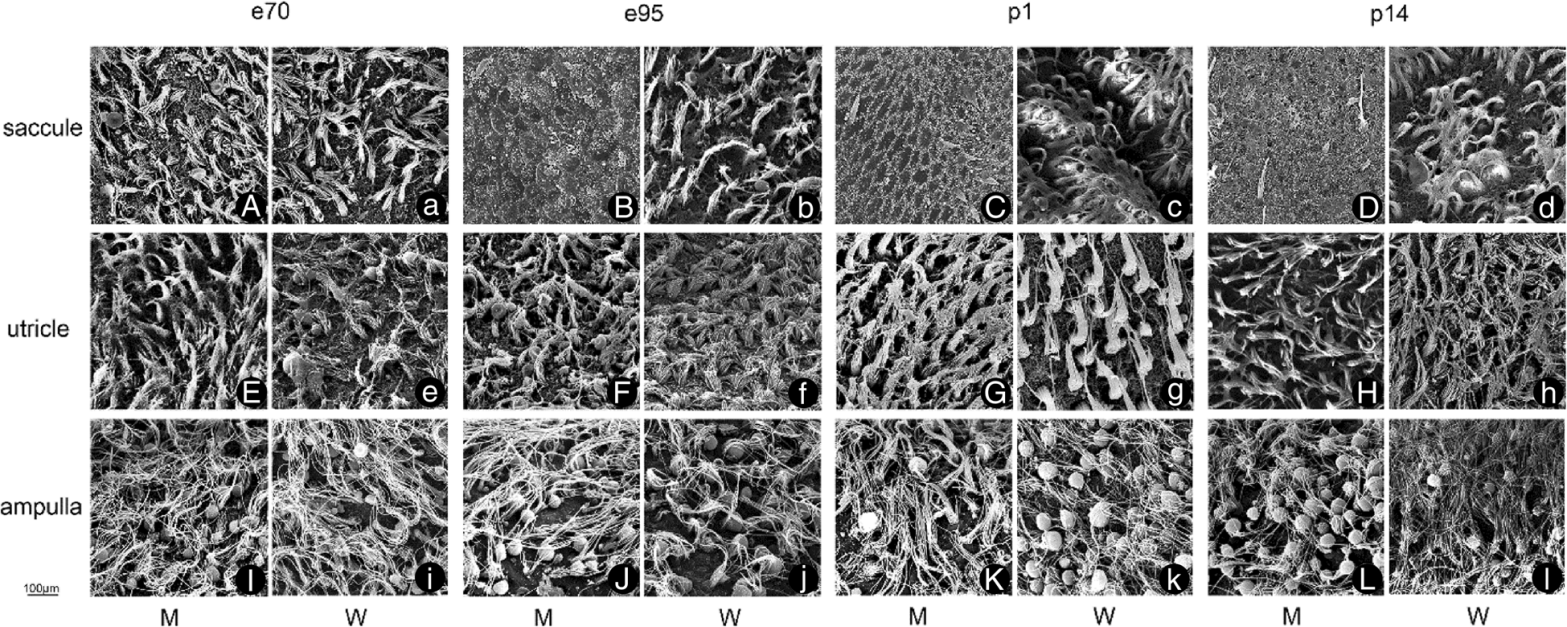
The morphological development of vestibular hair cells under scanning electron microscopy at embryonic day 70 (E70) and day 95 (E95), postnatal days 1 and day 14 (P14) (Majuscule **a**-**i** is from mutation type, lowercase a-I is from wild type). The comparison showed that mutation type has extensive saccule hair cell loss at E95, P1, and P14. However, most hair cells were still present at E70

Severe collapsed saccular hair cells in MITF-M mutation model was observed on postnatal 14 days. To investigate whether this degeneration was inherent, we checked four critical developmental time points, i.e., E70, E95, P1 and P14 (Fig. 6). Similar to P14, widespread vestibular hair cell loss was also noticeable in E95 and P1 compared to wild type. However, the saccular hair cells at E70 were intact and countable. This result indicated that hair cell damage happened in the process of cellular development. No hair cell damage was observed in the utricle and semicircular canal during the whole developmental period (E70 to P14).

### The vestibular function of miniature pigs

#### VEMP result

**Fig. 7:**
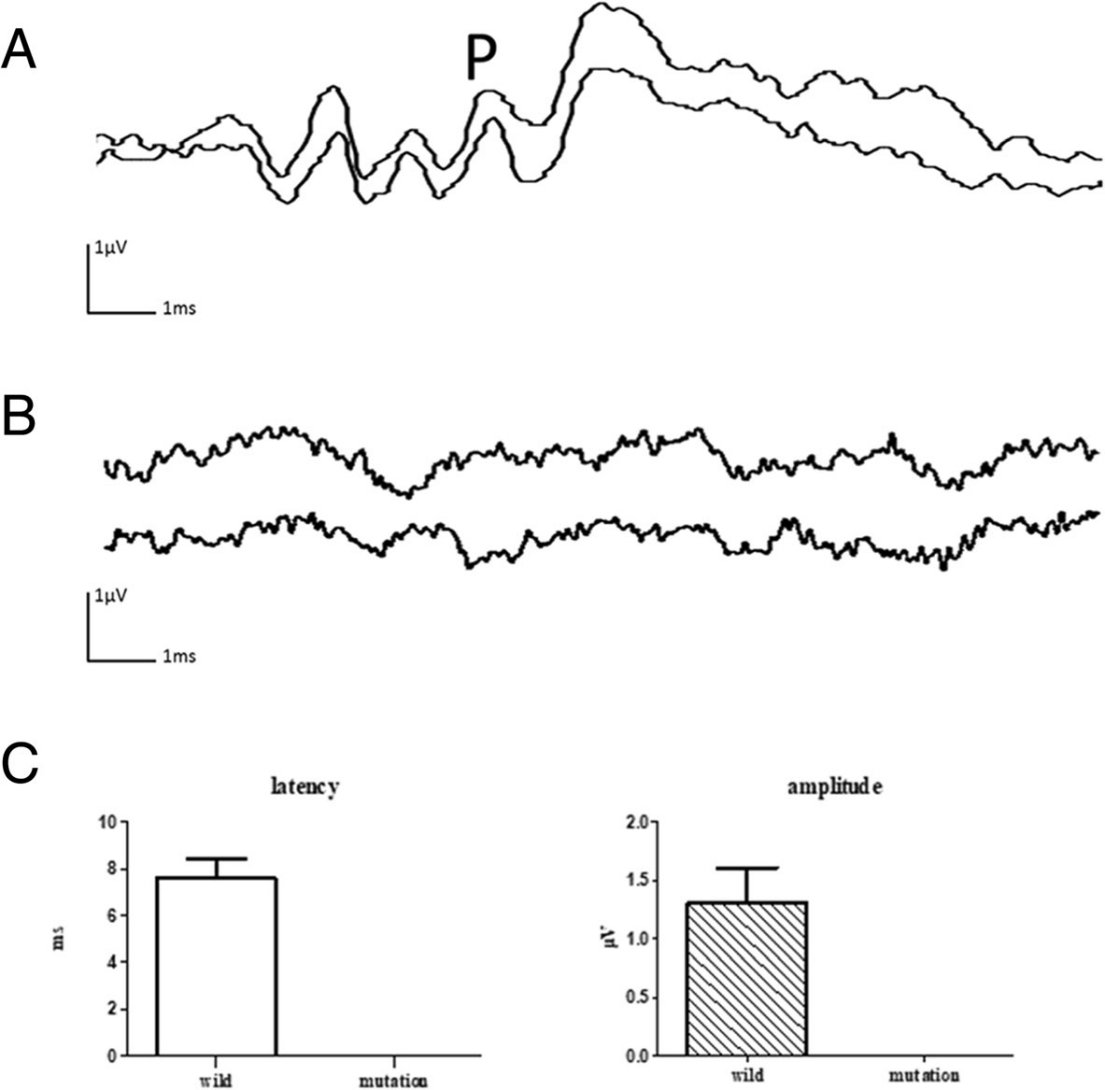
Electromyographic response recorded from the miniature pig’s masseter muscle from Wild type (**a**) and MITF-mutant (**b**) pigs during acoustic stimulation of the saccule. **c** illustrated the pooled data of both mutation and wild pigs

In the WT miniature pig, the latency of wave P of VEMPs recorded from the masseter muscles was 7.60 ± 0.78 ms, with an average amplitude of 1.31 ± 0.28 μV. The reduction rate of the VEMP amplitude was 100, 75 and 100% at 100, 90, and 80 dB SPL, respectively. However, mutant pig showed absent evoked potentials. The threshold of VEMP was 80 dB SPL (Fig. 7).

#### Locomotion result

Before gentamicin treatment, WT miniature pig showed normal balance behavior with no tilt and no body sway. After unilateral injection of gentamicin into the left middle ear, pig started to show tilt motion toward the left side (damaged side) and engendered a circling trajectory. This scenario may be the result of vestibular hair cell damage after exposure to gentamicin [22] which caused vertigo. Albino pig after gentamicin treatment showed a similar vertigo behavior and trajectory during locomotion. Injection of gentamicin into left ear caused anti-clockwise sway. This test suggested that the surviving ampulla hair cells in albino pig were functional.

## Discussion

We reported a spontaneous hearing loss model found in pigs. The mutation was found in a non-regulatory region of the melanocyte-specific promoter of microphthalmia-associated transcription factor gene (Mitf). It caused early degeneration of intermediate cells of cochlear SV and a profound hearing loss [5]. In this paper, we studied the effect of MITF-M gene mutation on vestibular development. We detected substantial damage of saccular hair cells in MITF-M mutation pigs under SEM micrograph. Consistently, no VEMP response was discovered in the porcine model, suggesting impaired function of saccule. Interestingly, the saccular hair cell loss was still present at the age of E70, suggesting this hair cell damage was not inherent, but caused during development. MIFT-M gene mutation did not affect hair cells in the utricle or semi-circular canals or bony labyrinth.

### MITF gene played a vital role in WS2 mammals, including humans [5]

**Fig. 8:**
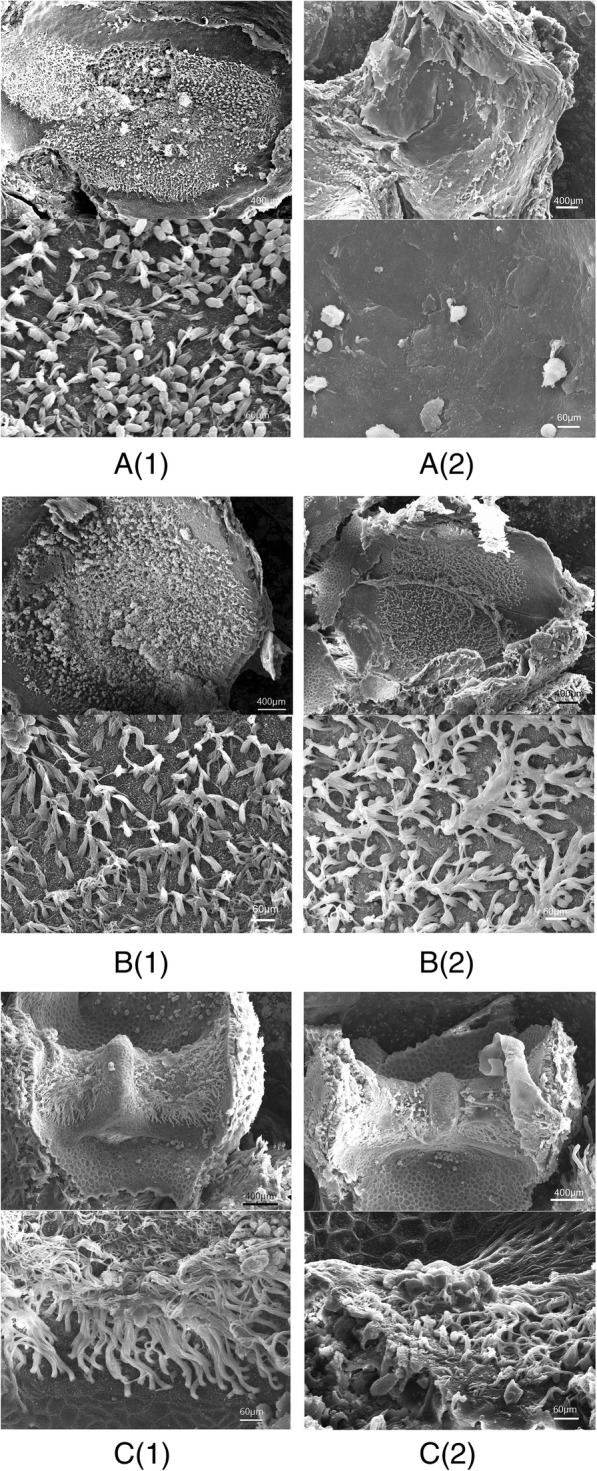
Scanning electron micrographs of saccule, utricle and crista ampulla in postnatal three weeks mice. **a** (1): saccule image from wild mouse (upper: low magnification image; below: high magnification image); **a** (2):saccule image from MITF mouse with extensive hair cell loss throughout the sensory epithelium (upper: low magnification image; below: high magnification image); **b** (1): utricle image from wild mouse (upper: low magnification image; below: high magnification image); **b** (2): utricle image from MITF mouse with numerous hair cells throughout the sensory epithelium (upper: low magnification image; below: high magnification image); **c** (1): crista ampulla image from wild mouse (upper: low magnification image; below: high magnification image); **c** (2):crista ampulla image from MITF mouse with numerous hair cells throughout the sensory epithelium (upper: low magnification image; below: high magnification image). Scale bars = 400 μm in upper images, 60 μm in below images

Our findings were consistent with this result which suggesting WS not only caused hearing loss but also affected vestibular organs. Using this porcine mutation model, our research provided strong evidence that MITF-M gene mutation could cause WS and impair the development of saccule, without interfering utricle or semicircular canals. To verify the appearance of MITF-M gene mutation in vestibular organs, we used SEM micrograph in 3 weeks mice to demonstrate various development. Similar severe hair cell loss was evident in saccule in MITF mouse (Fig. 8).

Stria vascularis (SV) and vestibular dark cells are engaged in inner ear fluid production [23], whose homeostasis is essential for normal function of hearing and balance. Why is saccule the only affected component in the vestibular system? As is well known, the high concentration of potassium in the cochlear part is maintained by the marginal cells of the SV. SV is also responsible for generating endocochlear potential (EP), the driving force for hair cells to transduce mechanical stimuli into electrical signals [24]. Recent studies showed that the K^+^ channels and intermediate cells were vital in EP and K^+^ transport [25, 26]. Our previous research indicated that the lack of intermediate cells was notable in the SV of albino pigs [5], which would influence endolymphatic potential [23]. The reduction of K^+^ influx into endolymph caused the decline of resting membrane potential, which would lead to Ca^2+^ leaking in, resulting in the depolarization of the hair cells, and eventually would trigger Ca^2+^ − mediated apoptosis [24]. Potassium circulation in the vestibular system (dark cells section) involved the same process as cochlea except the fact that intermediate cells formerly did not exist in vestibular endorgans [23]. Our results suggested the survival of cochlear hair cells and saccular hair cells was dependent on the potassium release from the cochlear SV, but hair cells of the utricle and semi-circular canals were independent on the SV. It should be noted that cochlear and vestibular labyrinths may have independent systems of endolymph secretion [27]. Neither dark cells nor marginal cells existed in the saccular wall, and the source of high potassium was usually considered to be the cochlea through the ductus reuniens [28]. Dark cells were found in all vestibular endorgans except saccule, which was consistent in guinea pig, bat, opossum, cat, Rhesus monkey and man [27].

Our animal model also has potential values in studying the role of otolith in the detection and transduction of head-directional signals. Saccule damage may affect compensatory movements in the neck and the limbs because targets of the saccular nerve activation were the neck, forelimb, back muscle and cervical inter motoneurons [29]. Moreover, vestibular stimulation modulated the formation of the hippocampus by diminishing the activity of head-direction cells and place cells [30]. The absence of such stimulation may result in spatial memory deficits [31] and selective atrophy of the hippocampus [32, 33].

As SEM provides a relevant “surface” view of the sensory epithelia [34, 35], vestibular nerves, whose function and integrity were meaningful and noteworthy, should be further investigated in our further research. With the concern of only 3% of the saccular-activated neurons had direct ascending branches to the oculomotor nucleus [29], the VOR analysis which was not included in this article should also be discussed after that.

## Methods

### Animals

Chinese Rongchang pigs were aging between embryonic day 47 to postnatal two months, from the animal breeding facility of Chongqing Academy of Animal Science, were used in this study. Adult pigs (P-6 m) were chosen to conduct the vestibular function test. Albino pigs with MITF-M mutation were characterized by missing their black ring around eyes (Fig. 1b) and heterochromia irides (Fig. 1d). Normal Rongchang pigs were used as the control group (Fig. 1a). The study was conducted with the approval of the Institutional Animal Care and Use Committee of General Hospital of PLA.

### Vestibular function test

#### Otolith function test

Vestibular evoked myogenic potentials (VEMPs), commonly used to objectively evaluate the saccular function of both human and animals, were recorded in two 0.5-year-old miniature pigs (one WT and one albino pig) [20]. The gait, head swings, trunk twisting, and circling were observed in both the WT and mutate pigs to detect vestibular disorders. For the VEMP test, pigs were anesthetized with 3% pentobarbital sodium (1 ml/kg) and Sumianxin II (0.1 ml/kg). As the masseter muscles are easier to be surgically exposed than the neck extensor muscles, we recorded the response of masseter muscles for VEMP test [21]. VEMP response was elicited by open field sound stimuli. The recording electrode was inserted into the lower third masseter muscles, and the reference electrode was inserted in the tip of the nose. The ground electrode was inserted in the scalp of the vertex. A rubber ball was placed between the upper and lower incisors to open the jaw to activate the masseter muscle. The response of VEMPs was evoked by tone-burst at 1 kHz (5 times per second) through ER3A earphone inserted in the auditory canal. The myogenic signal was amplified 100 k with a band-pass filter (30–1000 Hz) and was recorded using Smart-EP software (Intelligent Hearing Systems, Miami, USA). The signal was averaged 128 times with an analysis window of 50 ms. The VEMP was evoked by sound stimulus at 100 dB SPL which decreased at 10 dB step until the waveform disappeared. Two consecutive recordings were collected at threshold levels to verify repeatability and stability. Three more pigs in each group were tested to confirm the results.

#### Locomotion test

Effect of intratympanic injection of gentamicin (in one ear) on locomotion was observed in one WT pig and one albino pig. As gentamicin can quickly be diffused into inner ear through the round window membrane, it can diminish peripheral vestibular function. A single intratympanic application of gentamicin could result in a severe reduction in the spontaneous vestibular activity [22] and could lead to abnormal rotational behavior. In our study, the test was performed in a light room where pigs were allowed to roam freely in a 2 m diameter circle. Three more pigs in each group were tested to confirm the results.

### Morphological assessment of vestibular organs

#### Micro-CT scanning and 3D reconstruction

For morphological assessment, animals were sacrificed using an overdose of urethane (1.5 g kg^− 1^). The left temporal bones of the pigs (wild-type (WT) pigs (*n* = 4) and albino pigs(*n* = 4)) were dissected to expose the middle and inner ears. All samples were scanned using a high-speed Micro-CT system (PerkinElmer Inc., Norwalk, CT, USA) with the voxel size of 39 μm. Then MIMICS (Materialises interactive medical image control system, Belgium) was used for 3D image construction.

#### Sample preparation for scanning electron mcroscopy (SEM)

Pigs at the age of postnatal 14 days (one WT and one albino pig) were chosen to investigate the anatomy of vestibular organ affected by MITF-M mutation. Each pig was sacrificed under general anesthesia by inhalation of ethyl ether. All left inner ears of the samples were fixed with 2.5% glutaraldehyde in 0.1 M sodium cacodylate buffer (pH 7.4) containing 2 mM CaCl_2_. The samples were then washed with PBS solution and fixed afterward for 15 min in PBS solution which contained 1% OsO_4_. After dehydration in a serial of graded ethanol (up to 100%), the samples were dried and mounted on aluminum stubs. Then they were sputter-coated with gold particles and examined under the scanning electron microscope (Hitachi S-3700 N, Hitachi, Tokyo, Japan). Three more pigs in each group were tested to confirm the results. All the samples were carefully collected and incinerated after this research.

## Conclusions

In summary, a WS model with collapsed saccular hair cells in miniature pigs was established in this article. This research indicated that the major vestibular performance of this WS model was severe saccule hypofunction (no recordable VEMPs). Hair cells in other vestibular organs, i.e., the utricle and semicircular canals, were well-developed. However, the damage in saccular hair cells was not inherent but caused during the embryonic stage of development. Given the morphology and clinical performance of saccule detriment, the clinical diagnosis of otolith function should be considered in WS patients. Our results can help to interpret clinical balance disorders in WS patients.

## Abbreviations

CT: Computed Tomography
MITF-M: microphthalmia-associated transcription factor-M
